# Pruritus

**Published:** 1876-06

**Authors:** 


Pruritus.—The author of this short notice states that recently he had a most obstinate and severe case of pruritus of the vulva, clitoris, and mons veneris of a fat woman more than sixty years of age. So severe was it that she would cry from the distress. It so overcame her, every way, that she was nearly helpless. After trying a great number of remedies, both internally and externally, for several months, with very little relief, at length, with only the external application of the essence of peppermint to the itching parts, she was almost instantly relieved, with an occasional use of the same.—*Med. and Surg. Reporter.*

